# Stability-Indicating HPLC Determination of Gemcitabine in Pharmaceutical Formulations

**DOI:** 10.1155/2015/862592

**Published:** 2015-03-08

**Authors:** Rahul Singh, Ashok K. Shakya, Rajashri Naik, Naeem Shalan

**Affiliations:** ^1^Faculty of Pharmacy, Integral University, Kursi Road, Lucknow 226-026, India; ^2^Faculty of Pharmacy and Medical Sciences, Al-Ahliyya Amman University, P.O. Box 263, Amman 19328, Jordan

## Abstract

A simple, sensitive, inexpensive, and rapid stability indicating high performance liquid chromatographic method has been developed for determination of gemcitabine in injectable dosage forms using theophylline as internal standard. Chromatographic separation was achieved on a Phenomenex Luna C-18 column (250 mm × 4.6 mm; 5*μ*) with a mobile phase consisting of 90% water and 10% acetonitrile (pH 7.00 ± 0.05). The signals of gemcitabine and theophylline were recorded at 275 nm. Calibration curves were linear in the concentration range of 0.5–50 *μ*g/mL. The correlation coefficient was 0.999 or higher. The limit of detection and limit of quantitation were 0.1498 and 0.4541 *μ*g/mL, respectively. The inter- and intraday precision were less than 2%. Accuracy of the method ranged from 100.2% to 100.4%. Stability studies indicate that the drug was stable to sunlight and UV light. The drug gives 6 different hydrolytic products under alkaline stress and 3 in acidic condition. Aqueous and oxidative stress conditions also degrade the drug. Degradation was higher in the alkaline condition compared to other stress conditions. The robustness of the methods was evaluated using design of experiments. Validation reveals that the proposed method is specific, accurate, precise, reliable, robust, reproducible, and suitable for the quantitative analysis.

## 1. Introduction 

**Figure 1:**
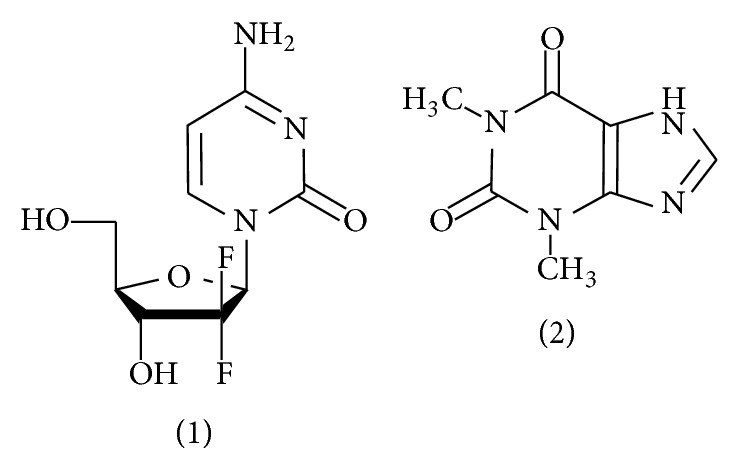
Chemical structures of gemcitabine (1) and theophylline (2).

Gemcitabine hydrochloride (Figure 1), (4-amino-1-[(2R, 4R, 5R)-3, 3-difluoro-4-hydroxy-5-(hydroxymethyl) oxolan-2-yl] pyrimidin-2-one) is a *β*-difluoronucleoside, purine antimetabolite. The drug is an antitumor agent, employed extensively against several human malignancies like ovarian, lung, pancreatic, bladder, urothelial, and breast cancer. It is currently marketed as a lyophilized powder. The drug is also extensively employed as antiviral agent, enzyme inhibitor, immunosuppressive agent, and radiation-sensitizing agents. Gemcitabine is a prodrug that enters the cell by means of nucleoside transporters and becomes active through an intracellular transformation catalyzed by deoxycytidine kinase to its diphosphate and triphosphate derivatives. The triphosphate derivative is incorporated into the DNA strand, inhibiting thymidylate synthetase which inhibits DNA synthesis and chain elongation, contributing to the antineoplastic activity of the drug. The diphosphate derivative inhibits ribonucleotide reductase, the enzyme responsible for catalyzing synthesis of deoxynucleoside-triphosphate required for DNA synthesis. Gemcitabine triphosphate competes with endogenous nucleoside triphosphate for incorporation into DNA [1–3].

A literature survey reveals that only a few methods based on ultraviolet spectroscopy [4], HPTLC [5], and HPLC [6–13] are available for determination of drug in formulation. Although several HPLC [14–22] and LC-MS/MS [23–28] methods have been reported for estimation of drug and its metabolites in biological fluids, these methods [23–28] are complicated, costly, and time consuming in comparison to a simple HPLC-UV method. A few stability indicating that HPLC methods [3, 11, 12] have been reported, which provides variable level of degradation of gemcitabine. Jansen et al. [3] reported the separation and identification of degraded product of gemcitabine in acidic stress condition. Mastanamma et al. [11] and Kudikala et al. [12] have reported the validated stability indicating method which can separate the hydrolytic degraded product of gemcitabine. However, to the best of our knowledge none of the HPLC method reported the oxidative degraded product of gemcitabine. Previously published methods for formulation are less robust and need more investigations for method development and validation. Stability-indicating methods have to demonstrate that they are specific, which involves evaluating the drug in the presence of its degradation products [29]. The present investigation describes a simple, rapid, accurate, precise, robust stability indicating RP-HPLC method for the determination of gemcitabine for dosage forms. The robustness of the method was studied using 2^4^ factorial design. The method was validated as per the ICH guidelines.

## 2. Experimental

### 2.1. Chemicals and Reagents

The reference sample of gemcitabine was supplied by M/s Shilpa Medicare Limited, Raichur, India. Theophylline (2) was received as gift sample from Hetero Pharmaceutical Ltd., Hyderabad, India. The marketed formulation of drug (Cytogem, Dr. Reddys, Mumbai, India) was purchased from the local market. All reagents were of analytical grade unless stated otherwise. Reverse osmosis quality water (purified with a Milli-RO plus Milli-Q station Millipore Corp., USA) and HPLC quality water were used throughout. Acetonitrile and methanol were supplied by Panreac (Barcelona, Spain).

### 2.2. HPLC Instrumentation and Conditions

A Shimadzu Prominence high pressure liquid chromatographic instrument provided with a Luna C-18 column (250 mm × 4.6 mm; 5 *µ*), an LC 20AT-VP solvent delivery system, a universal loop injector (Rheodyne 7725 i) of injection capacity of 20 *µ*L, and an SPD 20A UV-visible detector (*λ*_max⁡_ 275 nm) was employed in the study. Data acquisition was carried out using LC-Solution software. Chromatographic analyses were carried out using the mobile phase of acetonitrile-water (10 : 90; pH adjusted to 7.0 using trietylamine and orthophosphoric acid). The mobile phase was prepared daily and filtered through a 0.45 *µ*m membrane filter (Millipore Corp., USA). The temperature of column was maintained at 25 ± 1°C. Robustness, cross-validation and stability studies were carried out on Shimadzu Prominence Liquid Chromatographic system consists of quaternary gradient pump: Shimadzu-20-AD UFLC; Degasser: DGU-20A3 Prominence Degasser; Autosampler and Injector: SIL-20A Prominence Autosampler; Detector: Diode Array Detector (SPD-M20A); Communication Bus module: CBM-20A; and Column: Luna C-18 column (250 mm × 4.6 mm; 5 *µ*). The signals were captured using Windows based LC-Solution software (version 1.25).

### 2.3. Preparation of Stock and Standards Solutions

#### 2.3.1. Gemcitabine Standard and Working Solutions

An accurately weighed amount (100 mg) of gemcitabine was transferred into 100 mL calibrated flask and dissolved in appropriate volume of methanol. Then, the void volume was completed with methanol to produce a stock solution of 1000 *μ*g/mL. The stock solution was further diluted with mobile phase to obtain working solutions (25, 100, and 200 *μ*g/mL).

#### 2.3.2. Preparation of the Internal Standard (IS) Solution

An accurately weighed amount (100 mg) of theophylline was transferred into 100 mL volumetric flask and dissolved in 25 mL of methanol. The resultant solution was thoroughly sonicated till complete dissolution of the drug has occurred. Volume was made up to the mark with water.

#### 2.3.3. Calibration Standards

Calibration standards were prepared freshly using either stock or intermediate working solution of gemcitabine and internal standard. Standard solution of concentrations 0.5, 1.0, 2.0, 5.0, 10, 15, 25, 40, and 50 *µ*g/mL was prepared. All these solutions were containing 20 *µ*g/mL of theophylline as standards. These solutions were analyzed immediately to avoid degradation.

#### 2.3.4. Quality Control Samples

Similarly, quality control samples of concentration 1, 5, 20, 30, and 45 *µ*g/mL containing IS (20 *µ*g/mL) were prepared freshly and analyzed.

### 2.4. Preparation of Sample for Assay

Twelve injection vials containing the drug in the lyophilized powder form of two different batches were studied. Their aluminum closures were removed. The vials were weighed with the drug and after removing the content in empty state. With the help of the data available weight of the lyophilized powder was calculated. An accurately weighed portion from this powder equivalent to 10 mg of drug was transferred to 100 mL calibrated volumetric flask and 10 mL of internal standard solution was also transferred to it quantitatively. 50 mL of methanol was added to the flask and the contents of the flask were swirled, sonicated for 5 min, and then completed to volume with water. The prepared solutions were diluted quantitatively with mobile phase to obtain a solution of 20 *μ*g/mL drug and internal standard for the analysis.

### 2.5. Analytical Method Validation

#### 2.5.1. Linearity, Limit of Detection (LOD), and Limit of Quantitation (LOQ)

Appropriate volumes of gemcitabine stock standard solution (1000 mg/mL) were diluted with mobile phase to produce concentrations of 0.5, 1.0, 2.0, 5.0, 10, 15, 25, 40, and 50 *µ*g/mL. Replicates of each concentration were independently prepared and injected into the chromatograph. Linearity was evaluated by the linear least-squares regression model using weighting factor *x*. Microsoft office excel 2007 was used for statistical analysis. The method was validated according to ICH guidelines of the validation of analytical methods [29]. A 5% significance level was used for evaluation. The method was evaluated by determination of the correlation coefficient and intercept values. LOD and LOQ were determined from the best fitted calibration curve. LOD and LOQ were calculated as 3.3 × *σ*_*n*−1_/*S* and 10 × *σ*_*n*−1_/*S*, where *σ*_*n*−1_ is the standard deviation of the intercept and *S* is the slope of the calibration curve.

#### 2.5.2. Precision

Precision was measured using triplicate determination of quality control samples of 1, 5, 20, 30, and 45 *µ*g/mL of gemcitabine, on different occasions (0, 3, and 6 h) and different days. The precision (RSD) of the method was determined as intraday precision (repeatability) and intermediate precision. The intermediate precision was estimated from the RSD of the analysis of the samples prepared at the same concentration but on 3 different days at different concentration levels, while intraday precision was calculated by analyzing the same concentration during the same day at different time.

#### 2.5.3. Accuracy

Accuracy of method was determined by addition of known amounts of gemcitabine (*n* = 3, at each level of 25, 50, 80, 100, and 120% levels) to a sample solution of known concentration (formulation). From these solutions appropriate solution was prepared and analyzed and the total amount recovered was calculated. In this work, the mean recovery of the target concentration was 100 ± 2% for acceptance.

#### 2.5.4. Robustness

It is a measure of reproducibility of test results under normal, expected operational condition from analyst to analyst. The robustness of the method was evaluated on the basis of precision, as measured by percent coefficient of variation (RSD), determined as each concentration level was required not to exceed 2%. Design of experiments was used to study robustness of the method. A 2^4^ factorial design was used to test the robustness of chromatographic separation. The experimental design is useful for this kind of study as it facilitates the investigation of several parameters by reducing the number of experiments. Acetonitrile content of the mobile phase, pH, column oven temperature, and flow rate was investigated. Upper and lower limits are shown in Table 5. The experiments were run randomly with sample containing gemcitabine and internal standard (20 *µ*g/mL of each). The selected responses were resolution (*R*_*s*_), tailing factor of drug (*T*_*f*_-D), and tailing factor of IS (*T*_*f*_-I).

#### 2.5.5. Stability Studies

Stress study like oxidative, alkaline, and acidic stress, exposure to sunlight and UV light (254 nm), was carried out using raw material. Chromatograms were recorded in order to study the specificity of the method. The chromatograms of the samples were compared with those of control samples that were freshly prepared from the stock standard solution and without stress. All samples were analyzed in triplicate. The peak purity was checked using the tools of the LC-Solution software. This assessment was based on the comparison of spectra recorded during the elution of the peak. UV spectra and peak purity were used to assess purity of analytes.

*(1) Oxidative Stress. *Gemcitabine (5 mg) was weighed accurately and transferred to 100 mL flask for evaluation of oxidative stress. 10 mL of 5% hydrogen peroxide was added to it. It was shaken for one hour at 60°C and then contents were cooled to room temperature. Internal standard (2 mL) was added; the contents were transferred quantitatively to 100 mL volumetric flask and diluted to the mark with mobile phase.

*(2) Effect of Acidic, Alkaline, and Aqueous Media. *Similarly for evaluation of acidic, alkaline, or hydrolytic stress, gemcitabine (5 mg) was weighed accurately and transferred to 100 mL flask. These samples were shaken (at 60°C, 1 h) with either hydrochloric acid (5 mL, 1 N HCl) or sodium hydroxide (5 mL, 1 N NaOH or 20 mL water). After one hour the content was cooled and processed as described above (in oxidative stress). 

*(3) Effect of UV Light or Sunlight.* Gemcitabine (500 mg) was placed in an open watch glass and exposed to either UV-irradiation (~100 W/m^2^) or direct sunlight for one hour with occasionally shifting of the content using stainless steel spatula. After exposure, 5 mg of sample was weighed and transferred to 100 mL volumetric flask; internal standard (2 mL) was added to it and further processed as described above.

Chromatograms of these sample solutions were recorded and compared with the chromatograms of unexposed API.

*(4) Stock Stability. *The stability of stock solution was evaluated at zero time and stored in the refrigerator (2–8°C). Samples were prepared and analyzed at days 0, 7, 14, and 21.

## 3. Results and Discussion

### 3.1. Analytical Method Development

**Figure 2:**
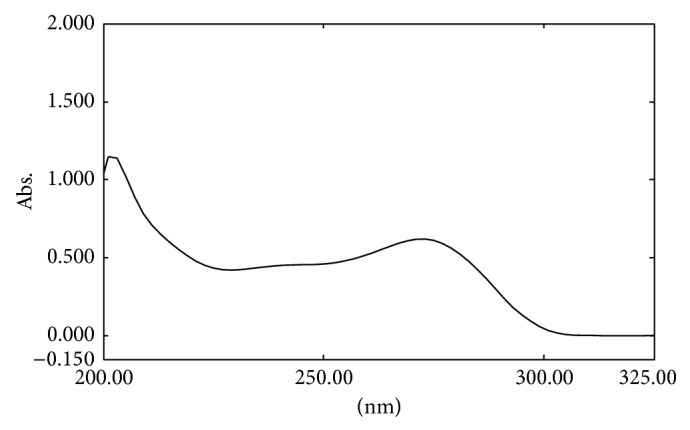
UV spectra of the gemcitabine in mobile phase.

**Figure 3:**
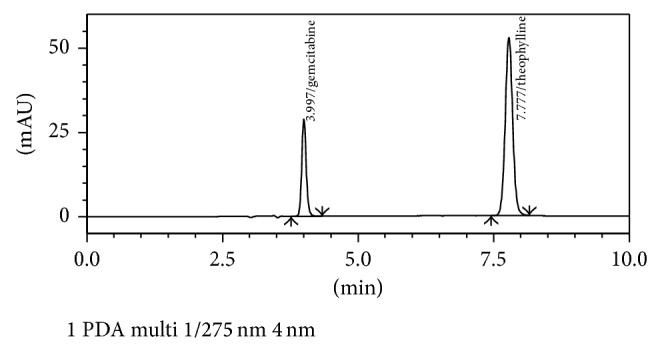
Representative chromatogram showing signals of gemcitabine and theophylline in the selected mobile phase.

In order to achieve optimum separation various parameters like solvent, solvent strength, detection wavelength, flow rate, elution time, asymmetry, and plate numbers were considered. During optimization gemcitabine hydrochloride and internal standard were injected into various mobile phases of water : methanol or water : acetonitrile (90 : 10, 80 : 20, 70 : 30, 60 :  40, and 50 : 50, pH 5, 6, or 7) and the retention time, tailing factor along with resolution factor, was recorded. In certain mobile phases the peak was distorted while in others the compound eluted out quickly indicating the lesser retention time and thus lesser separation on the column. As the pKa of gemcitabine is 3.5 and unstable in an acidic pH [3], different mobile phases of pH 7.0 were used. Mobile phase of acetonitrile-water (10 : 90, v/v, pH adjusted to 7.0) was used as suitable mobile phase, as it was able to separate the analytes. Using the C-18 ODS analytical column with an isocratic mobile phase at a flow rate of 1 mL/min, the drug and IS were eluted at ~4.0 and 7.8 min, respectively. Although temperature was found not to be a critical parameter for this analysis, it was set at 25°C to avoid shifting of signals. The absorption maximum of the drug at 275 nm was selected for detection (Figure 2), as there was no interference from excipients present in drug or baseline disturbance. The resolution factor was ~11.  Figure 3 depicts the representative chromatogram obtained with the present method.

### 3.2. Method Validation

The method was validated with respect to parameters including linearity, limit of quantitation (LOQ), limit of detection (LOD), precision, accuracy, specificity, robustness, system suitability, and stability.

#### 3.2.1. Linearity

Different calibration curves (*n* = 6) that were constructed for gemcitabine were linear over the concentration range of 0.5–50 *µ*g/mL. Peak area ratios of gemcitabine to IS were plotted versus gemcitabine concentration and linear regression was performed using Spinchrome-Clarity or LC-solution software. Different calibration curves (*n* = 6) were prepared on three different days. The mean regression equation for gemcitabine was found to be *y* = 0.0353*x* + 0.0063 with 0.9998 correlation coefficient, using weighting factor-*x* (Table 1). The linearity range reported in other methods ranged between 0.020 and 300 *µ*g/mL [3, 5–28].

#### 3.2.2. LOD and LOQ

The LOD and LOQ values were 0.1498 and 0.4541 *µ*g/mL calculated using calibration curve as per ICH guideline. The LOD and LOQ reported by Lanz et al. [14] were 10 and 20 ng/mL, based on signal to noise ratio method, while Xu et al. [7] reported 12 and 37.5 ng/mL based on calibration curve (external standard method).

#### 3.2.3. Accuracy and Precision

The accuracy and precision of the analytical method were established across its linear range as indicated in the guideline. As shown from the data in Table 2, excellent recoveries were made at different added concentration level. The results obtained for the intraday and interday precision of the method, expressed as RSD values. As shown in the table, the intraday and interday RSD were <2.0% for all concentrations tested in different situations studied (Tables 2 and 3).

#### 3.2.4. Specificity

Specificity of the method was assessed by comparing the chromatograms obtained from lyophilized powder, from internal standard, and from the drug standards. The retention times of drug from standard solutions and from lyophilized powder were identical and no coeluting peaks from the diluents were observed, indicating specific method for quantitative estimation of drug in the commercial formulation.

#### 3.2.5. System Suitability

System suitability parameters were studied with six replicates standard solution of the drug and the calculated parameters are within the acceptance criteria. The tailing factor, the number of theoretical plates, and HETP were in the acceptable limits (RSD less than 2%). The system suitability results are shown in Table 4.

#### 3.2.6. Robustness

**Figure 4:**
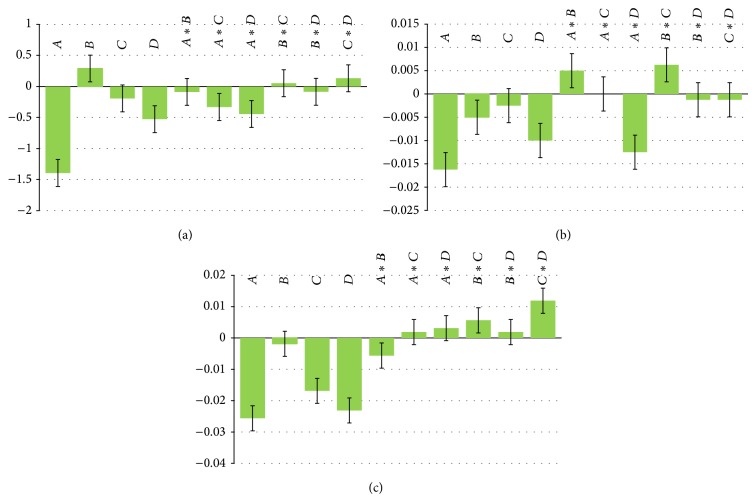
Scaled and centered coefficient of variation (%) of (a) resolution factor, (b) tailing factor of drug, and (c) tailing factor of I.S. during robustness studies.

Robustness of the methods was illustrated by getting the resolution factor and tailing factor, when mobile phase flow rate (±0.2 mL/min), acetonitrile content (±2%), pH (±0.2 units), and column temperature (±5°C) were deliberately varied. It was studied using factorial design experiment. The deliberate changes in the method do not affected the resolution, tailing factor of drug, and IS significantly. The scaled and centered coefficient plots for the above responses revealed that different parameters did not affect responses, so that the developed method was considered rugged and robust. Results are presented in Figure 4 and Table 5.

#### 3.2.7. Stability Studies

**Figure 5:**
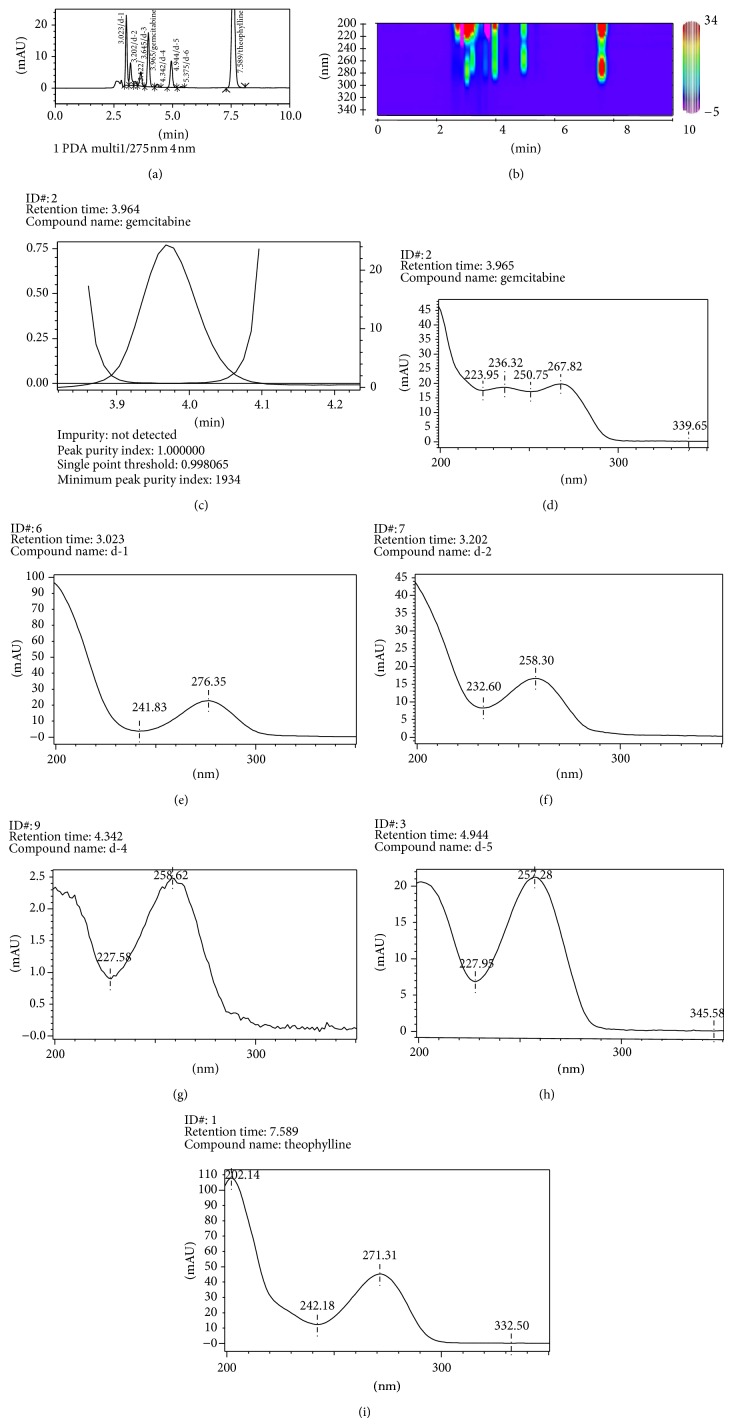
Typical HPLC chromatogram of (a) gemcitabine exposed to alkaline stress (1 N NaOH, 60°C, 1 h), (b) contour plot, (c) peak purity index, and extracted UV spectra of (d) gemcitabine, (e) degraded product d-1, (f) d-2, (g) d-4, (h) d-5, and (i) theophylline.

**Figure 6:**
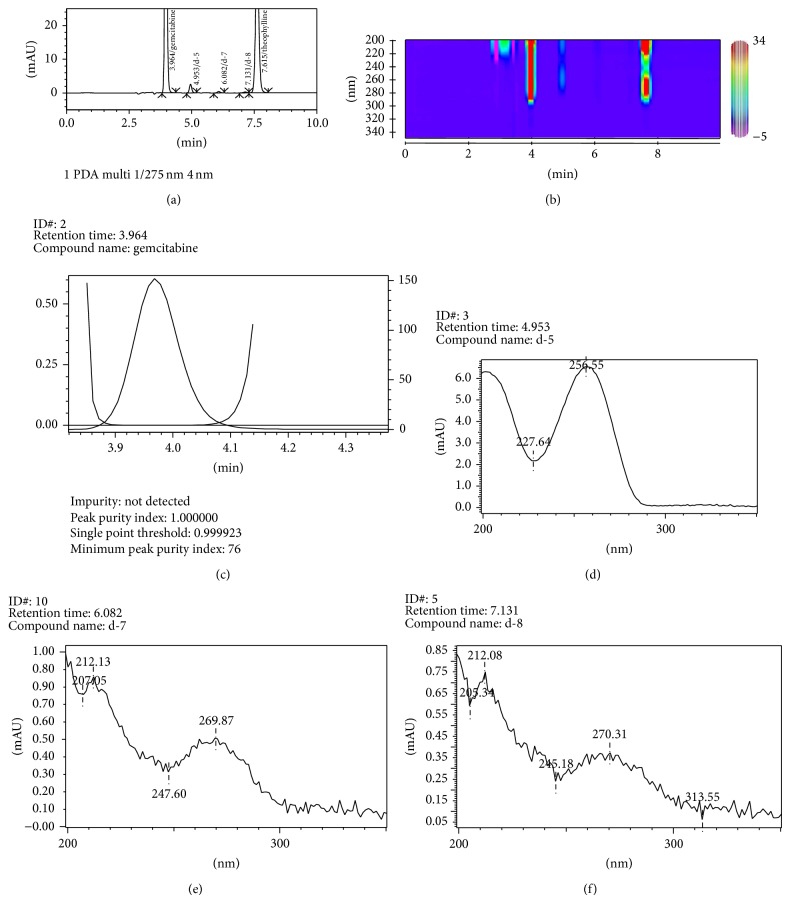
Typical HPLC chromatogram of (a) gemcitabine exposed to acidic stress (1 N HCl, 60°C, 1 h), (b) contour plot, (c) peak purity index, and extracted UV spectrum of (d) degraded product d-5, (e) d-7, and (f) d-8.

**Figure 7:**
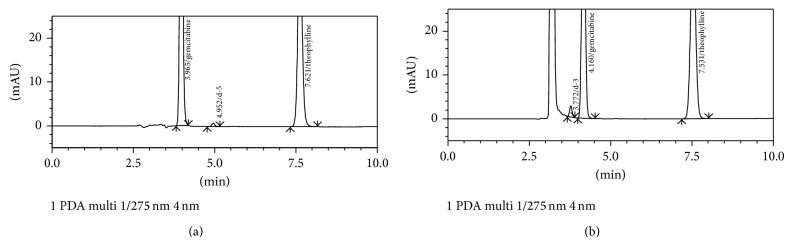
Typical HPLC chromatogram of gemcitabine exposed to (a) hydrolytic (H_2_O, 60°C, 1 h) and (b) oxidative stress (5%, 60°C, 1 h).

The prepared stock and working solutions were stable up to 21 days when stored in refrigerator (2–8°C) and did not produce degraded compounds during experimental conditions. The peak purity was 0.985 or more during the validation studies. Gemcitabine produces six different degradation products on alkaline stress with retention time (min) 3.023 (as d-1), 3.202 (d-2), 3.645 (d-3), 4.342 (d-4), 4.944 (d-5), and 5.375 (d-6). The percentage of gemcitabine remained was 16.1%. Mastanamma et al. [11] and Kudikala et al. [12] have reported two degraded product of gemcitabine.  Figure 5 represents the chromatogram, contour plot, peak purity, and UV spectra of gemcitabine and degraded products. The extracted UV spectra indicate that the entire degradation products are derived from gemcitabine or its intermediates. In case of acidic stress the degradation products were observed at 4.953 (as d-5), 6.082 (d-7), and 7.131 min (d-8) (Figure 6). Jansen et al. [3] have reported the presence of a coeluted degraded product with gemcitabine which does not possess UV absorption at 275 nm. The hydrolytic product d-5 (4.95 min) was observed in alkaline as well as in aqueous stress condition. On exposure to hydrogen peroxide (5%, 60°C, 1 h), gemcitabine produces only one minor degradation products having retention time 3.772 min (Table 6, Figure 7). The percentage of unoxidized gemcitabine was 97.1%. Gemcitabine was completely degraded on exposure to drastic oxidative condition (50%, 60°C, 1 h). However, these degraded compounds have no ultraviolet (UV) absorbance at 275 nm, the wavelength used to monitor the gemcitabine concentrations. Borisagar et al. [5] reported the oxidative degradation [13.8%] of gemcitabine utilizing HPTLC. The present results indicate that, using appropriate chromatographic conditions, the structurally related degraded products of gemcitabine can be separated which were not studied earlier (Table 8).

#### 3.2.8. Assay

The proposed method was applied to the determination gemcitabine in injectable formulations. The results of these assay yielded 99.6% (RSD, 0.26%) of labeled claimed. Low value of precision indicates that the method can be used precisely for the estimation of drug in formulations (Table 7).

## 4. Conclusion

A validated HPLC method has been developed for determination of gemcitabine in formulations. The proposed stability indicating method is simple, economical, accurate, precise, specific, and robust. Method is capable of separating different degraded products of drug which can be estimated separately. The experimental design was found to be very useful in testing the robustness of chromatographic separation during the validation step. Hence this method can be easily and conveniently adopted for the routine analysis of gemcitabine in pharmaceutical dosage form.

## Figures and Tables

**Table 1:** Linearity data of the proposed method.

Conc. (*µ*g/mL)	Mean peak area (gemcitabine) (*n* = 6)	Mean peak area (IS)	Mean area ratio	Conc. found (*µ*g/mL)	% assay
0.5	11556.2	490138.6	0.02358	0.50	99.0
1.0	22196.2	527242.0	0.04210	1.03	102.6
2.0	40119.6	516202.8	0.07772	2.05	102.3
5.0	99206.8	549256.6	0.18062	4.99	99.9
10.0	197702.2	546987.8	0.36144	10.18	101.8
15.0	298096.4	551610.8	0.54041	15.30	102.0
25.0	496084.0	560316.6	0.88536	25.19	100.8
40.0	791722.0	566686.4	1.39711	39.85	99.6
50.0	987580.6	553478.0	1.78432	50.95	101.9
*y* = 0.0353*x* + 0.0063, *r* ^2^ = 0.9998					

**Table 2:** Accuracy of the method.

Amount taken (*µ*g/mL)	Amount added		Amount recovered (mean ± SD) (*n* = 6)	% recovery (mean ± SD)	RSD
	%	*µ*g/mL			
20	25	5	25.09 ± 0.10	100.37 ± 0.40	0.40
20	50	10	30.06 ± 0.09	100.19 ± 0.31	0.31
20	80	16	36.15 ± 0.21	100.42 ± 0.59	0.59
20	100	20	40.14 ± 0.13	100.34 ± 0.33	0.33
20	120	24	44.07 ± 0.06	100.15 ± 0.13	0.13

**Table 3:** Precision study of the proposed method.

Concentration (*µ*g/mL)	Intraday precision		Interday precision	
	Conc. found	RSD	Conc. found	RSD
	Mean ± SD		Mean ± SD	
1	0.994 ± 0.015	1.52	0.985 ± 0.018	1.81
5	5.030 ± 0.074	1.48	5.041 ± 0.092	1.83
20	19.920 ± 0.243	1.22	19.840 ± 0.280	1.41
30	29.886 ± 0.292	0.98	29.861 ± 0.233	0.78
45	44.759 ± 0.255	0.57	44.742 ± 0.326	0.73

**Table 4:** System suitability.

Parameters	Mean	RSD
Theoretical plates (Drug)	7716	1.25
Plates/meter	30864	1.25
HETP	31.10	0.13
Tailing factor	1.10	0.11
LOD (*µ*g/mL)	0.1498	1.05
LOQ (*µ*g/mL)	0.4541	1.05
Resolution (Rs)	11.0	0.45
Retention time of drug	3.95 min	1.50
Retention time of IS	7.80 min	1.50

**(a):** 

Selected parameters and their variations	−1 (lower limit)	+1 (upper limit)
Acetonitrile in mobile phase (%) (*A*)	8	12
Final pH of the mobile phase (*B*)	6.8	7.2
Column oven temperature (°C) (*C*)	20	30
Flow rate (mL/min) (*D*)	0.8	1.2

**(b):** 

Exp. number	Run order	*A*	*B*	*C*	*D*	*R*_*s*_	*T*_*f*_-D	*T*_*f*_-I
1	6	8	6.8	20	0.8	14.01	1.12	1.2
2	11	12	6.8	20	0.8	13.1	1.11	1.19
3	5	8	7.2	20	0.8	14.1	1.09	1.18
4	13	12	7.2	20	0.8	13.2	1.08	1.1
5	1	8	6.8	30	0.8	14.3	1.09	1.11
6	7	12	6.8	30	0.8	10.2	1.09	1.08
7	10	8	7.2	30	0.8	14.5	1.11	1.15
8	15	12	7.2	30	0.8	11.85	1.1	1.08
9	12	8	6.8	20	1.2	13.51	1.13	1.11
10	14	12	6.8	20	1.2	10.21	1.05	1.07
11	9	8	7.2	20	1.2	14.41	1.09	1.12
12	2	12	7.2	20	1.2	10.05	1.06	1.07
13	8	8	6.8	30	1.2	13.25	1.12	1.1
14	4	12	6.8	30	1.2	10.15	1.04	1.07
15	3	8	7.2	30	1.2	14.12	1.1	1.11
16	16	12	7.2	30	1.2	10.15	1.05	1.05

*R*_*s*_: resolution factor, *T*_*f*_-D: tailing factor for drug, and *T*_*f*_-I: tailing factor for IS.

**Table 6:** Stability data under different stressed conditions.

Stress conditions	Percent gemcitabine remained	Retention time of degraded products
Alkaline stress (1 N, NaOH, 60°C, 1 h)	16.1 ± 0.2%	3.023 (d-1), 3.202 (d-2), 3.645 (d-3), 4.342 (d-4), 4.944 (d-5), and 5.375 (d-6)
Acidic stress (1 N HCl, 60°C, 1 h)	95.0 ± 0.2%	4.953 (d-5), 6.082 (d-7), and 7.131 (d-8)
Oxidative stress (5%, 60°C, 1 h)	97.1 ± 0.1%	3.772 (d-3)
Aqueous hydrolytic stress (60°C, 1 h)	99.2 ± 0.1%	4.952 (d-5)
Ultraviolet light (100 W/m^2^, 1 h)	100.0%	0.0
Direct sunlight (1 h)	100.0%	0.0
Aqueous stability (after 21 days)	99.9 ± 0.1%	0.0

**Table 7:** Assay of formulations.

Sample	Label claim (mg/vial) (*n* = 6)	Amount found	% assay	% RSD
		Mean ± SD		
Batch 1	200	199.35 ± 0.46	99.7	0.23
Batch 2	200	199.27 ± 0.52	99.6	0.26

**Table 8:** Comparison between analytical methods.

S. number	Analytical method (reference)	Drugs	Column	Detection (*λ*_max⁡_)	Silent features and advantages.	Disadvantage
1	HPLC-PDA (Jansen et al., 2000) [3]	Gemcitabine	Zorbax C-8 (5 *μ*m, 4.6 × 250 mm)	275 nm	Sensitive, simple method applicable for separation of drug and degraded products. Degradated products were identified using spectroscopy.	Gradient elution. Only the degradated products of acidic stress studies were separated. Run time: 20 min.

2	HPLC (Rao et al., 2007) [6]	Gemcitabine	ODS column (5 *µ*m, 4.6 × 250 mm)	234 nm	Linearity range: 1–300 *µ*g/mL.	High organic waste (70% acetonitrile). Stability studies not performed.

3	HPLC (Xu et al., 2014) [7]	Gemcitabine and curcumin	Phenomenex C-18 (5 *μ*m, 4.6 × 250 mm)	270 nm (Gem.); 420 nm (curcumin)	LOD: 0.012 *µ*g/mL LOQ: 0.038 *µ*g/mL. Linearity range: 0.038–1.5 *µ*g/mL. Sensitive, accurate, and precise method, developed using design of experiment.	Gradient elution, narrow range of linearity, run time: 15 min. Nonstability indicating method (effect of pH and oxidation, exposure to sunlight or UV light, was not studied).

4	HPLC (Mastanamma et al., 2010) [11]	Gemcitabine	C-18 (5 *μ*m, 4.6 × 250 mm)	270 nm	Linearity range: 10–60 *µ*g/mL. Simple and rapid stability indicating method.	Organic waste (40%, methanol) and low sensitivity.

5	HPLC (Kudikala et al., 2014) [12]	Gemcitabine	Enable C18G column (5 *µ*m, 4.6 × 250 mm)	285 nm	LOQ: 1 *µ*g/mL. Linearity range: 1–45 *µ*g/mL. Simple stability indicating method.	Hydrolytic (aq.) and oxidative degraded products not studied. Gemcitabine shows high tailing factor.

6	HPLC (Devanaboyina et al., 2014) [13]	Gemcitabine	Kromasil (5 *µ*m, 4.6 × 150 mm)	247 nm	Linearity range: 50–300 *µ*g/mL.	High organic waste (30% acetonitrile). Stability studies not performed.

7	HPLC (Rajesh et al., 2011) [10]	Gemcitabine Capecitabine	Intersil 3, C-18 column (5 *µ*m, 4.6 × 150 mm)	260 nm	Linearity range: 10–50 *µ*g/mL.	High organic waste (70% acetonitrile). Stability studies not studied.

8	HPLC (Lanz et al., 2007) [14]	Gemcitabine	C18 (3 *µ*m particle size, 4.6 mm × 100 mm)	276 nm	LOQ: 0.02 *µ*g/mL. Linearity range: 0.02–20 *µ*g/mL. Sensitive method for analysis of drug in plasma and serum.	Gradient elution, run time: 17 min. Applicable only for serum and plasma samples. Effect of pH, oxidation, or light on stability of raw material/formulation is not studied.

9	LC-MS-MS (Nussbaumer et al., 2010) [28]	Gemcitabine and other anticancer drugs	—	Mass spectrometry (MS-MS)	LOQ: 0.25. Linearity range: 0.25–2 ng/mL. Sensitive, simple method applicable for detection of several drugs.	Lower accuracy (85–110%) and precision (15%). Applicable for bioequivalence and pharmacokinetic studies where 15% precision is permitted. Gradient elution, run time: 21 min.

10	HPTLC (Borisagar et al., 2012) [5]	Gemcitabine	—	268 nm	Linearity range: 500–3000 ng/spot. Rapid HPTLC method for analysis. Stability indicating method.	—

11	Proposed HPLC method	Gemcitabine	Phenomenex Luna C-18 column (5 *µ*m, 4.6 × 250 mm)	275 nm	LOD: 0.15 *µ*g/mL. Linearity ranges from 0.5 to 50 *µ*g/mL. Isocratic, economical (less organic waste), efficient stability indicating method. Capable of separating different hydrolytic and oxidative products of drug which can be estimated separately. Symmetrical peak shape.	Degraded products are separated but not quantized.
